# Mechanical suppression of osteolytic bone metastases in advanced breast cancer patients: a randomised controlled study protocol evaluating safety, feasibility and preliminary efficacy of exercise as a targeted medicine

**DOI:** 10.1186/s13063-018-3091-8

**Published:** 2018-12-20

**Authors:** Nicolas H. Hart, Daniel A. Galvão, Christobel Saunders, Dennis R. Taaffe, Kynan T. Feeney, Nigel A. Spry, Daphne Tsoi, Hilary Martin, Raphael Chee, Tim Clay, Andrew D. Redfern, Robert U. Newton

**Affiliations:** 1 Exercise Medicine Research Institute, Edith Cowan University, 270 Joondalup Drive, Joondalup, Perth, Western Australia 6027 Australia; 2 Institute for Health Research, University of Notre Dame Australia, Perth, WA Australia; 3 School of Medical and Health Sciences, Edith Cowan University, Perth, WA Australia; 4 St John of God Hospital, Perth, WA Australia; 5 Royal Perth Hospital, Perth, WA Australia; 6 School of Medicine, University of Western Australia, Perth, WA Australia; 7 School of Medicine, University of Notre Dame Australia, Perth, WA Australia; 8 Genesis CancerCare, Perth, WA Australia; 9 Fiona Stanley Hospital, Perth, WA Australia; 10 School of Human Movement and Nutrition Sciences, University of Queensland, Brisbane, QLD Australia

**Keywords:** Tumour suppression, Tumour growth, Resistance training, Aerobic training, Isometric training, Muscle activity, Exercise medicine, Advanced cancer, Bone metastases

## Abstract

**Background:**

Skeletal metastases present a major challenge for clinicians, representing an advanced and typically incurable stage of cancer. Bone is also the most common location for metastatic breast carcinoma, with skeletal lesions identified in over 80% of patients with advanced breast cancer. Preclinical models have demonstrated the ability of mechanical stimulation to suppress tumour formation and promote skeletal preservation at bone sites with osteolytic lesions, generating modulatory interference of tumour-driven bone remodelling. Preclinical studies have also demonstrated anti-cancer effects through exercise by minimising tumour hypoxia, normalising tumour vasculature and increasing tumoural blood perfusion. This study proposes to explore the promising role of targeted exercise to suppress tumour growth while concomitantly delivering broader health benefits in patients with advanced breast cancer with osteolytic bone metastases.

**Methods:**

This single-blinded, two-armed, randomised and controlled pilot study aims to establish the safety, feasibility and efficacy of an individually tailored, modular multi-modal exercise programme incorporating spinal isometric training (targeted muscle contraction) in 40 women with advanced breast cancer and stable osteolytic spinal metastases. Participants will be randomly assigned to exercise or usual medical care. The intervention arm will receive a 3-month clinically supervised exercise programme, which if proven to be safe and efficacious will be offered to the control-arm patients following study completion. Primary endpoints (programme feasibility, safety, tolerance and adherence) and secondary endpoints (tumour morphology, serum tumour biomarkers, bone metabolism, inflammation, anthropometry, body composition, bone pain, physical function and patient-reported outcomes) will be measured at baseline and following the intervention.

**Discussion:**

Exercise medicine may positively alter tumour biology through numerous mechanical and non-mechanical mechanisms. This randomised controlled pilot trial will explore the preliminary effects of targeted exercise on tumour morphology and circulating metastatic tumour biomarkers using an osteolytic skeletal metastases model in patients with breast cancer. The study is principally aimed at establishing feasibility and safety. If proven to be safe and feasible, results from this study could have important implications for the delivery of this exercise programme to patients with advanced cancer and sclerotic skeletal metastases or with skeletal lesions present in haematological cancers (such as osteolytic lesions in multiple myeloma), for which future research is recommended.

**Trial registration:**

anzctr.org.au, ACTRN-12616001368426. Registered on 4 October 2016.

**Electronic supplementary material:**

The online version of this article (10.1186/s13063-018-3091-8) contains supplementary material, which is available to authorized users.

## Background

Bone metastases are generally incurable and clinically problematic, yet are present in over 80% of patients with advanced breast cancer [1, 2], representing a debilitating stage of disease with very poor patient prognoses during palliation, and one of the leading causes of breast cancer mortality among women worldwide [3–5]. Osteolytic (lytic) bone metastases, in particular, present a considerable challenge to patients and clinicians due to rapid microarchitectural deterioration of affected skeletal sites through tumour-driven dysregulation of bone metabolic activity in favour of excess resorption [1, 6, 7]. Metastatic breast carcinomas commonly deposit in trabecular (cancellous) regions of bone [6, 8–10], such as the skull, ribs, spine, pelvis and proximal and distal segments of long bones, owing to their strong affinity to red bone marrow [2, 7, 9, 11]. Of direct relevance, the majority of these skeletal structures are characteristically load-bearing [12–14], thus any accelerated bone loss and heightened fragility following tumoural infiltration [8, 10, 15–17] has immediate consequences for patients with cancer if left untreated or unmanaged. Consequently, this destructive skeletal process leads to increased patient morbidity and mortality, with heightened fragility, increased risk of spinal compression, potential development of hypercalcaemia, increased bone pain and increased fracture risk [7, 17–19]; whilst simultaneously creating a localised microenvironment conducive to tumoural growth and invasion within affected bone sites [20–23].

Chemotherapy, hormone therapy, radiotherapy and anti-resorptive medications are the primary therapeutic agents used in breast cancer palliation to delay disease progression, alleviate bone pain and associated symptoms, preserve skeletal integrity and extend survival [24–32]. Whilst effective, these treatments produce well-documented side effects to varying degrees, which can lead to dose limitation or cessation, subsequently restricting their full clinical utility. For example, patients with advanced breast cancer with bone metastases are provided with bone strengthening (anti-resorptive) medications such as bisphosphonates or denosumab to fortify bone through induced sclerosis leading to increments in bone density [33, 34]. While efficacious in the mid-term; long-term use of these medications themselves eventually generate skeletal fragility in the absence of a discontinuation period [35–37], which is not a clinically viable option for patients with advanced cancer and osteolytic bone metastases.

Bone is highly adaptive and osteogenically sensitive to its mechanical environment, principally through muscular contraction of neighbouring muscle, but also from impact and gravitational forces acting upon the skeleton during human movement and from external loads [12, 38, 39]. Up-regulation of osteoblastic activity through exercise may therefore allow mechanically driven regulation of bone metabolism (i.e. osteocyte-mediated coupling of osteoblast and osteoclast activity) to counteract tumour-driven dysregulation which could have two key benefits: (1) to preserve bone strength through anabolic morphological (material and structural) adaptations; and (2) to interfere with tumoural processes that blunt osteoblastic formation and promote osteoclastic resorption, thus potentially suppressing tumour growth in affected skeletal sites. Preclinical studies [8, 13, 40, 41] have explored this potential relationship in rodent orthotopic models, implanting human breast cancer cells into trabecular skeletal tissue in order to induce osteolysis in the load-bearing tibia. Impressively, when comparing loaded and non-loaded tumour-affected tibiae within host rodents, repeated bouts of externally controlled mechanical compression preserved skeletal integrity (0% versus 71% degradation for loaded and unloaded conditions), blunted tumour-mediated osteolysis, and significantly suppressed tumour growth by approximately 80% [8, 13] in the absence of muscular influence. In addition to osteocyte-driven osteogenic signalling, the inextricable anatomical, mechanical, metabolic and pleiotropic link between muscle and bone [12, 38, 42, 43] provides another avenue of therapeutic osteogenic and anti-tumour potential not yet explored [14, 44–47]. The voluntary activation of muscle surrounding skeletal lesions may deliver anabolic cytokines and myokines (secretome cross-talk) to lesion sites [14, 44, 45, 48] to countenance catabolic tumour-mediated processes [46, 47, 49, 50]. Accordingly, mechanical signals propagated by muscle contraction may concurrently stimulate osteocyte-mediated metabolic activity and myokine-cytokine secretome cross-talk to promote osteogenesis while modulating tumoural behaviour and biology in order to slow tumour growth [14, 45, 48–51].

Human clinical trials have yet to be implemented in this space, owing to a historical and misplaced belief that patients with advanced cancer and bone metastases should be excluded from exercise programmes and synonymous research due to increased risk of skeletal complications or other potential adverse events [52–54]. Formative work by Galvão and colleagues [51, 55–58] demonstrated the safety and feasibility of delivering supervised exercise to patients with advanced prostate cancer and bone metastases, avoiding exercises that placed direct or targeted stress on bones with identified lesions. Separate work by Rief and colleagues [59, 60] demonstrated the safety and feasibility of physiotherapy-instructed spinal isometric training in isolation for patients undergoing palliative radiation therapy for spinal bone metastases, though in a small cohort of heterogeneous patients with cancer and disparate lesion types. Taken together, these studies illustrate the potential for preclinical studies to be translated to human patients; specifically models of advanced breast cancer with osteolytic bone metastatic disease. This is particularly important as animal studies do not always translate to the human condition, particularly in exercise-mediated bone adaptation studies, often due to poorly designed human trials for equivalency [12, 61–63].

Given that bone metastases remain one of the leading causes of breast-cancer-related deaths worldwide, additional and novel interventions to target osteolytic lesions in skeletal tissue are highly clinically relevant. Expanding on our prior work, the aim of this study is to (1) assess the safety and feasibility of a supervised and individually tailored resistance, aerobic and flexibility exercise programme that includes targeted spinal isometric training in patients with advanced breast cancer and osteolytic bone metastases; (2) explore the preliminary efficacy of the exercise programme to slow tumour growth and tumour biomarker activity in target osteolytic spinal lesions and (3) examine the broader efficacy of the exercise programme to preserve muscle and bone mass, improve physical fitness, enhance physical function, reduce cancer-related fatigue and increase quality of life. It is the working hypothesis of this study that the exercise programme will be safe and feasible; will show signs of tumoural suppression and/or favourable alterations in tumour biomarkers; and will lead to positive physical and psychosocial outcomes for patients. If successful, the outcomes of this trial will be used to improve clinical knowledge pertaining to exercise prescriptions for patients with advanced cancer who have metastatic carcinomas and high disease burden, and will be used as a foundation for future phase II and phase III efficacy-focused human clinical trials to establish the anti-cancer effects of exercise. It is anticipated that this new information will aid in the establishment and/or renewal of clinical exercise guidelines for the management of cancer across the disease spectrum.

## Methods

### Study design

This single-blinded (investigators blinded to group allocation), two-armed, randomised and controlled (supervised exercise versus usual medical care) explorative clinical trial will examine the feasibility, safety and preliminary efficacy of combining targeted spinal isometric training with modulatory, multi-modal exercise (M3EP-SIT) in women with advanced breast cancer and stable osteolytic spinal bone metastases. The exercise group (intervention arm) will receive an individually tailored and supervised 12-week exercise programme involving resistance, aerobic, flexibility and isometric exercises in addition to usual medical care. The control group will receive usual medical care only during this time and will be asked not to change their baseline levels of physical activity. However, following the trial, the control group will be offered the same exercise programme if proven to be safe and efficacious. This procedure has been shown to be an effective strategy to minimise study contamination, patient withdrawal or loss of patients to follow up in prior exercise oncology trials [14, 52, 64–68].

### Recruitment

**Fig. 1:**
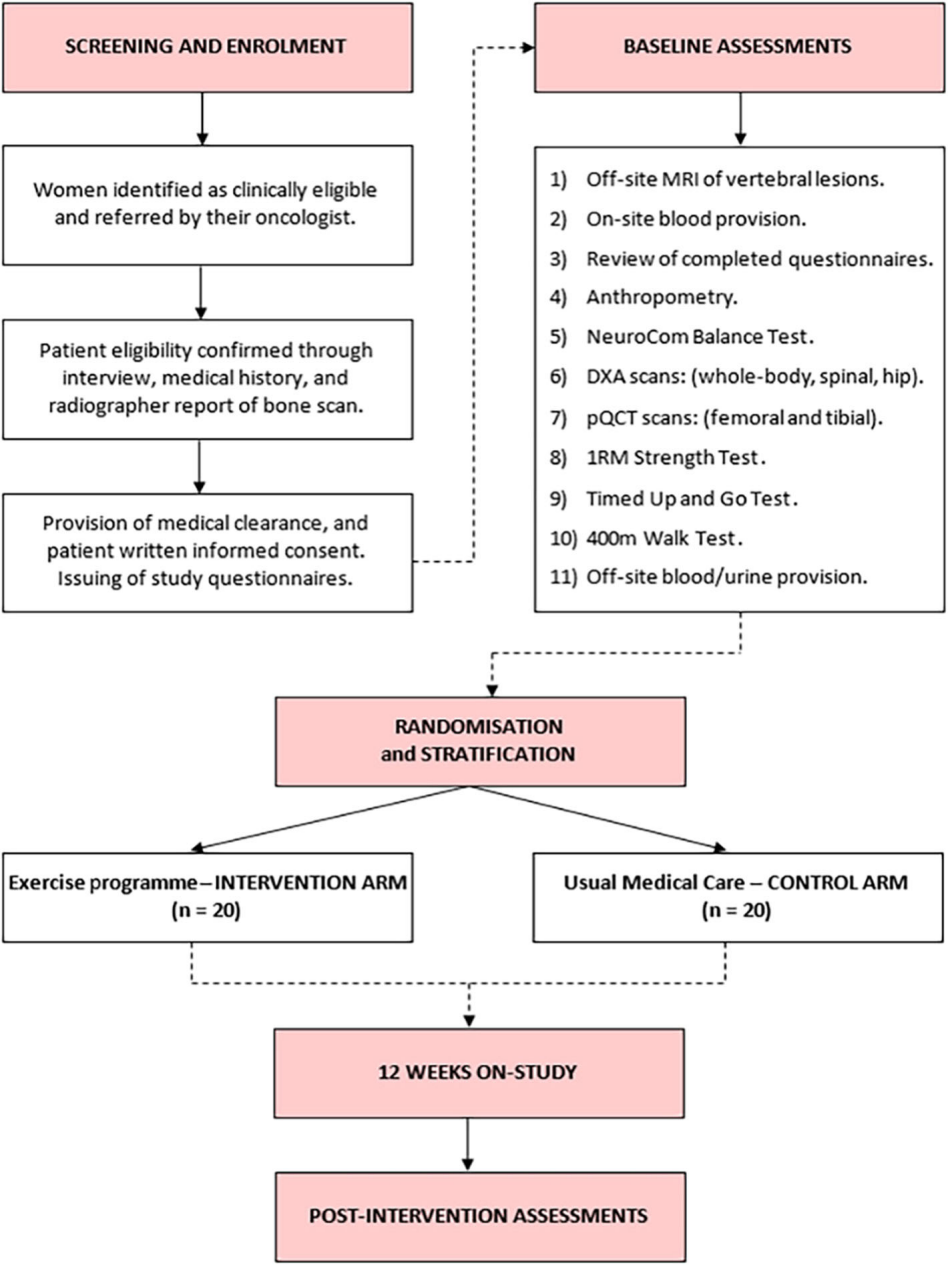
Schematic overview of the study protocol. MRI, magnetic resonance imaging; DXA, dual-energy x-ray absorptiometry; pQCT, peripheral quantitative computed tomography; 1RM, one-repetition maximum

Patients will be recruited by invitation of their cancer specialist (surgeon, radiation oncologist or medical oncologist) who will provide clinically eligible patients with a study information sheet and refer these patients to an independent study coordinator. If patients are interested in participation and their eligibility is confirmed, they will receive an informed consent document to read and sign in the presence of a study investigator and clinical research coordinator before undertaking baseline measurements prior to randomisation (Fig. 1).

### Randomisation

Patients will be randomly allocated in a ratio of 1:1 to the two study arms: exercise or usual care, stratified by age (≤ 60 years, > 60 years), hormone receptor (HR) status (HR+, HR-) [69] and time since completion of palliative radiotherapy to target spinal lesions (< 3 months, ≥ 3 months) and/or time since commencing or changing hormone therapy and/or chemotherapy (< 3 months, ≥ 3 months). A research officer with no patient contact will be responsible for randomisation of patients into either group using a computer-generated code through a random-assignment programme. Study investigators and exercise physiologists conducting testing procedures will be blinded to group allocation. Only exercise physiologists outside the research team will be permitted to deliver the exercise intervention to participants in order to maintain integrity of the blinding process.

### Participants

A total of 40 women (20 subjects per arm) with breast cancer and stable osteolytic bone metastases in cervical, thoracic and/or lumbar vertebrae will be invited to participate. They will be included if they have not engaged in regular exercise in the past 3 months (defined as undertaking structured aerobic and/or resistance training two or more times per week). Due to the novelty of this explorative clinical trial, our sample size is based on previous preclinical animal studies [13, 70–72], human pilot studies [14, 56, 58, 73, 74] and consideration of the ability to recruit patients with advanced breast cancer and osteolytic bone metastases during the trial. Specifically, to achieve 80% power at an alpha level of 0.05 (two-tailed), 16 subjects per group are required to demonstrate a meaningful difference (effect size ≥ 1.0) at the completion of the study for the primary endpoint, and most secondary endpoints. To account for an attrition rate of up to 25%, 40 subjects will be randomised equally between the study arms (exercise, *n* = 20; control, *n* = 20).

Patients are permitted to receive radiotherapy for non-spinal bone metastases while enrolled in this trial. Patients require medical clearance prior to enrolment, therefore must achieve an Eastern Cooperative Oncology Group (ECOG) performance status ≤ 1, and must not have any acute illness, significant bone pain or cardiovascular or neurological disorders that could inhibit exercise participation as judged by their managing physician. All participants must provide written informed consent prior to participation. Patients will be excluded from this trial if they are receiving experimental treatments. The protocol has been approved by the Human Research Ethics Committee (HREC) of Edith Cowan University (ECU), Project ID 14266 NEWTON; St John of God Hospitals (SJOG), Project ID 969 and Sir Charles Gairdner and Osborne Park Health Group (SCGOPHG), Project ID 2016–118. This trial is also registered with the Australia and New Zealand Clinical Trails Register (ANZCTR), Trial ID ACTRN-1261600136842.

### Measurements

Primary and secondary endpoints will be measured at baseline (week 0), post-intervention (week 13) and through-out the 12-week on-trial period (Table 1).

**Table 1:** Assessments of study feasibility

Measures	Time of collection
Recruitment and completion	
- Referred patients	Trial completion
- Eligible patients	Trial completion
- Enrolled patients	Trial completion
- Eligibility rate	Trial completion
- Recruitment rate	Trial completion
- Trial completions	Trial completion
- Patient withdrawals	Trial completion
- Patient drop-outs	Trial completion
- Trial contamination	Trial completion
Patient safety (control arm)	
- Number of adverse events	Tri-weekly record
- Severity of adverse events	Tri-weekly record
- Number of skeletal complications	Tri-weekly record
Patient safety (intervention arm)	
- Number of adverse events	At each exercise session
- Severity of adverse events	At each exercise session
- Number of skeletal complications	At each exercise session
Program tolerance (intervention arm)	
- Pre-sessional bone pain	At each exercise session
- Pre-sessional fatigue	At each exercise session
- Sessional rating of perceived exertion	At each exercise session
- Sessional tolerance	At each exercise session
Program adherence (intervention arm)	
- Number of completed sessions	Post intervention
- Number of missed sessions	Post intervention
Program compliance (intervention arm)	
- Prescribed vs. actual exercise completed (for each exercise mode).	Post intervention
- Percent of total volume completed (for each exercise mode).	Post intervention

#### Primary Endpoint

##### Feasibility

Study and programme feasibility will be quantified through a series of multi-item categories including patient recruitment and trial completion, patient safety and tolerance and program adherence and compliance (Table 2). Programme safety will be assessed by recording the incidence and severity (grading) of adverse events and/or skeletal complications [18] throughout the on-trial period for the intervention and control arms in accordance with the common terminology criteria for adverse events (CTCAE) v5.0 criteria. Skeletal complications will include heightened bone pain at sites of known bone metastases and/or pathological skeletal fractures. The nature, severity and impact of bone pain will be examined using the Functional Assessment of Chronic Illness Therapy (FACIT) bone pain questionnaire at baseline and post intervention.

**Table 2:** Schedule of assessments at baseline and post intervention

Measures	Baseline	Post intervention
Tumour morphology (off-site)		
- MRI: T1-axial	X	X
Tumour biomarkers		
- Blood: HIF-1α, TGF-β	X	X
Anthropometry		
- Height (cm)	X	
- Weight (kg)	X	X
- Waist circumference (cm)	X	X
- Hip circumference (cm)	X	X
- Femoral length (mm)	X	
- Tibial length (mm)	X	
- Body mass index (kg/m^2^)	X	X
- Waist-to-hip ratio	X	X
Body composition		
- DXA: whole-body, spinal, hip	X	X
- pQCT: femoral, tibial	X	X
Physical assessments		
- NeuroCom Balance Test	X	X
- 1RM Strength Test (Leg Extension)	X	X
- 400 m Walk Test	X	X
- Timed Up and Go Test	X	X
Other biomarkers (off-site)		
- Blood: P1NP, ALP, CRP, fasting glucose and lipids	X	X
- Urine: NTx	X	X
Questionnaires		
- Demographic and health history	X	
- Concomitant medications	X	X
- Health-related quality of Life (SF-36)	X	X
- Cancer-specific quality of life (EORTC: QLQ30, BR23)	X	X
- Bone Pain (FACIT-BP)	X	X
- Brief Symptom Index (BSI-18)	X	X
- Insomnia Severity Index (ISI)	X	X
- Godin Leisure-time Exercise	X	X
Exercise programme		
- Clinic exercise record sheet (prescribed vs. actual)	At each exercise session	
- Home exercise record sheet (prescribed vs. actual)	At each exercise session	
- Pre-session bone pain, muscle soreness and fatigue (VAS)	At each exercise session	
- Post-session rating of perceived exertion and tolerance (VAS)	At each exercise session	

*Abbreviations: HIF-1α* hypoxia-inducible factor 1-alpha, *TGF-β* transformation growth-like factor beta, *DXA* dual-energy x-ray absorptiometry, *pQCT* peripheral quantitative computed tomography, *P1NP* amino-terminal propeptide of type 1 procollagen, *NTx* amino-terminal collagen type-1 telopeptide, *ALP* alkaline phosphatase, *CRP* C-reactive protein, *SF-36* Short Form-36, *EORTC* European Organisation for Research and Treatment of Cancer, *FACIT-BP* Functional Assessment of Chronic Illness Therapy-Bone Pain, *VAS* visual analogue scale

Programme tolerance, adherence and compliance will be assessed in the intervention arm only. Specifically, programme tolerance will be quantified by routinely measuring pre-session bone pain, muscle soreness and general fatigue at each exercise session by visual analogue scale (VAS, 0–10) and by recording post-session rating of perceived exertion (Borg scale, 0–10) and post-session tolerance (VAS, 0–10) after each exercise session. Programme adherence and compliance will be assessed using an exercise diary completed by the patient at all clinic-based and home-based exercise sessions to record volume of resistance training (weight lifted (kilogrammes), sets and repetitions), aerobic training (intensity (level), duration (minutes), speed (revolutions per minute), heart rate (maximum and average) and rating of perceived exertion), flexibility training (repetitions and hold time (duration)) and isometric training (repetitions and hold time (duration)) completed. These data will be compared to the prescribed and individualised exercise programme provided to each patient in order to establish programme adherence (completed versus missed sessions) and compliance (prescribed versus actual exercise completed for each training mode (resistance, aerobic, flexibility and isometric)).

#### Secondary endpoints

##### Tumour morphology

Location of metastatic lesions will be initially identified through bone scans provided by the managing oncologist prior to referral to this study. Tumour morphology will be measured using axial T1-weighted magnetic resonance imaging (MRI) (1.5 T, Magnetom Essenza, Siemens, Victoria, Australia) in locations where osteolytic lesions have been identified in patients with bone metastases at either thoracic or lumbar spinal regions [75–78]. All patients will be scanned by the same radiologist using the same MRI machine and a standardised sequence and routine for scout and primary scan acquisition. Specifically, spinal bone metastases will be identified and confirmed using three preliminary axial scout scans in the sagittal plane to view the cervical, thoracic and lumbar regions, respectively (T2-weighted; imaging frequency = 63.66 Hz; slice thickness = 3.0–4.0 mm; spacing between slices = 3.6–6.0 mm; echo train length = 16–21; flip angle = 140–150°; acquisition matrix = 256\0\0\192 (cervical), 448\0\0\358 (thoracic) and 320\0\0\320 (lumbar)).

**Fig. 2:**
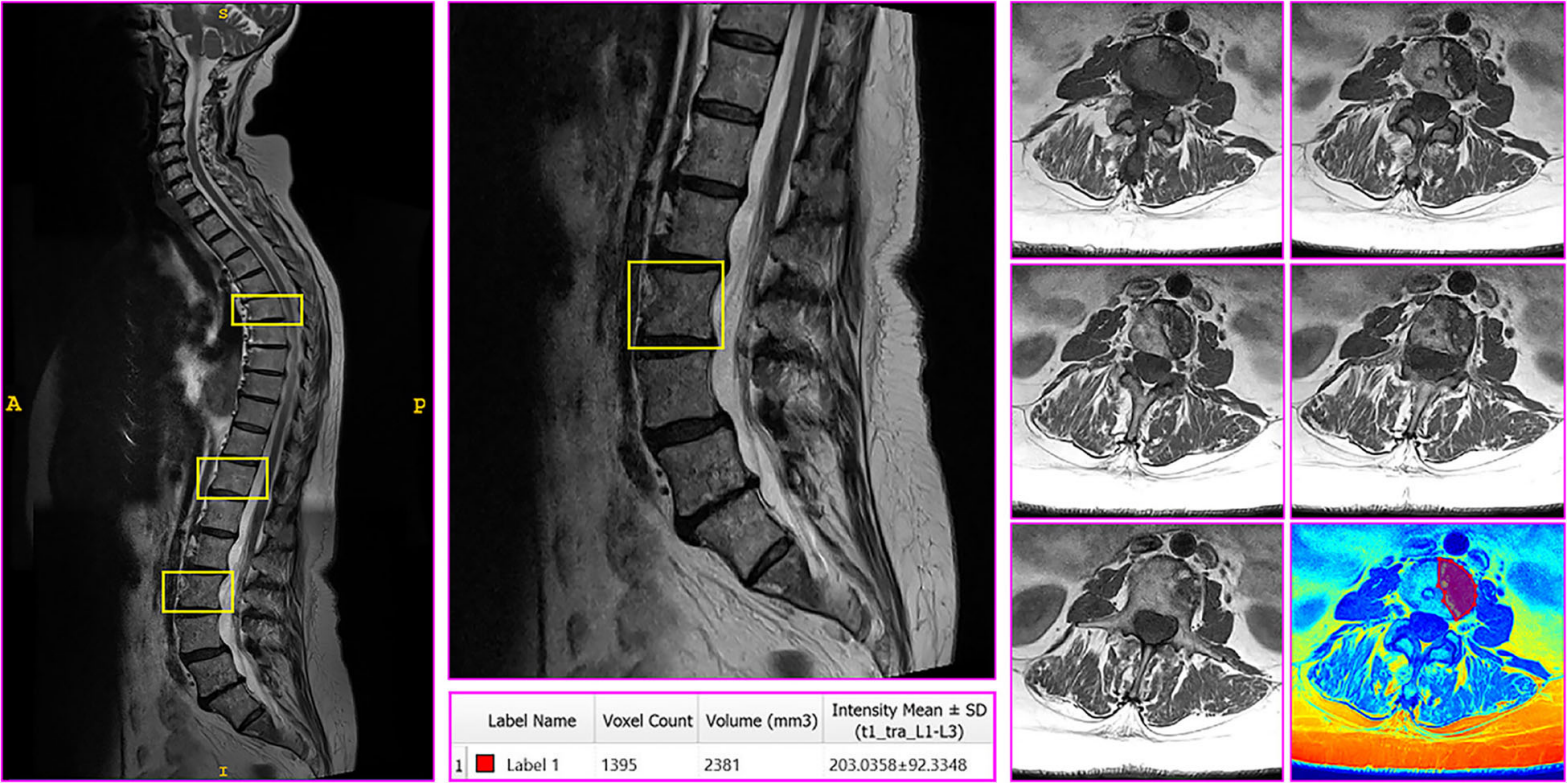
Example of magnetic resonance imaging (MRI) image acquisition. Left: cervical, thoracic and lumbar scout views stitched together with three osteolytic lesions identified at T5, T11 and L2. Top, middle: lumbar scout view at higher resolution with the L2 vertebra outlined. Bottom, middle: example data outputs provided by regional analysis. Right: slice by slice, cephalad to caudal, transverse view at each level of the L2 lesion, with an example colour map and tumoural analysis of one slice in isolation

Primary scans of each affected vertebra will be performed in the transverse plane, capturing the vertebrae above and below to produce an image with three vertebrae in total (T1-weighted; imaging frequency = 63.66 Hz; slice thickness = 4.0 mm; space between slices = 4.2 mm; echo train length = 3; flip angle = 150°; acquisition matrix = 384\0\0\307). Following the acquisition of images, tumour morphology (volume (cubic millimetres) and intensity (watts per steradian (W/sr))) will be examined for each slice using ITK-Snap (V3.6.0) image analysis software [79] (Fig. 2). All images will be examined at the conclusion of the study by two independent researchers for consistency in analysis and to establish intra-rater and inter-rater reliability coefficients.

##### Biomarkers

Metastatic tumour biomarkers, hypoxia-inducible factor 1-alpha (HIF-1α) and transformation growth-like factor beta (TGF-β) will be serologically examined to measure tumoural hypoxic activity and growth activity respectively; identified as synergistic drivers of metastatic tumour progression [45, 80–84]. Fasted serological and first-void samples for urianalysis will also be collected within 48 h of baseline and post-intervention testing sessions to measure bone metabolic activity and systemic inflammation. Specifically, bone formation marker, amino-terminal propeptide of type 1 procollagen (P1NP); bone resorption marker, amino-terminal collagen type-I telopeptide (NTx); bone disorder marker, alkaline phosphatase (ALP), inflammation marker, C-reactive protein (CRP) and fasting glucose and lipid profiles will be examined. All fasted serological and first-void biomarkers will be collected in the morning, and assessed by the same accredited laboratory (Australian Clinical Laboratories, Perth, Western Australia).

##### Anthropometry

Stature will be recorded to the nearest 0.1 cm using a wall-mounted stadiometer (Model 222, Seca, Hamburg, Germany), with body mass recorded to the nearest 0.1 kg using an electronic scale (AE Adams CPW Plus-200, Adam Equipment Inc., CT, USA), with body mass index (BMI) calculated as weight divided by height in metres squared (kg/m^2^). Waist and hip circumferences are defined as the mid-point between the 10th rib and the iliac crest and the level of the greater trochanter, respectively, with the waist-to-hip ratio calculated. Femoral length will be measured from the greater trochanter to the knee-joint axis and tibial length will be measured from the knee-joint axis to the medial malleolus. Waist circumference, hip circumference, femoral length and tibial length will be measured to the nearest 0.1 cm using a constant-tension, retractable measuring tape (Model 4414, Tech-Med Services, NY, USA). Stature, waist circumference and hip circumference will be measured in triplicate in each participant, with the average of each variable retained for analysis.

##### Musculoskeletal health

Whole-body, segmental (axial and appendicular) and regional (spinal and total hip) scans will be performed to examine bone area (BA), areal bone mineral content (aBMC), areal bone mineral density (aBMD) and lean mass using dual-energy x-ray absorptiometry (DXA; Hologic Discovery A, Waltham, MA, USA). Whole-body and appendicular segmentations will be analysed in accordance with Hart and colleagues [85]. Regional analyses (lumbar spine, total hip, femoral neck, trochanter, Wards triangle) will be performed in accordance with Hologic’s manufacturer specifications [86].

Appendicular, non-lesion control sites will be scanned to quantify bone material, structure and strength using peripheral quantitative computed tomography (pQCT; XCT-3000, Stratec, Pzochienheim, Germany). Specifically, trabecular, cortical, marrow and total volumetric density (Tb.vBMD, Ct.vBMD, Ma.vBMD, Tt.vBMD); trabecular, cortical, marrow and total cross-sectional area (Tb.Ar, Ct.Ar, Ma.Ar, Tt.Ar); cortical thickness (Ct.Th); stress-strain index (SSIPOL); absolute fracture load (FL.Ab) and relative fracture load (FL.Rel) of the left femur (4% and 33% slices) and left tibia (4%, 14%, 38% and 66% slices) will be measured and analysed in accordance with Hart and colleagues [87, 88]. Muscle cross-sectional area (Mu.Ar) will also be quantified.

##### Adiposity

Whole-body, segmental and central subcutaneous adipose tissue (fat mass), central visceral adipose tissue (VAT; area, mass and volume) and android-to-gynoid ratio will be measured using DXA. Whole-body and appendicular segmentations will be generated in accordance with Hart and Colleagues [85]. Fat area (Fa.Ar) and muscle density (Mu.Den) of the thigh and shank will be measured using pQCT [87, 88], as an indication of subcutaneous and intramuscular fat infiltration, respectively.

##### Objective measures of physical function

Muscle strength, aerobic capacity and physical function will be assessed. Muscle strength will be measured using the one repetition maximum (1RM) test for the leg extension exercise. This exercise was chosen as it can be safely performed by all patients included in this study. The 400 m walk test and Timed Up and Go test will be used as measures for aerobic capacity and physical function, respectively. In addition, patients will also undergo a comprehensive balance test (NeuroCom Smart Balance, Natus Medical Inc., USA).

##### Quality of life, anxiety, distress, insomnia and physical activity

Health-related quality of life outcomes for general health, pain, vitality, social functioning, emotional role and mental health will be measured using the Short Form 36 (SF-36, IQOLA) survey. In addition, the European Organisation for Research and Treatment of Cancer (EORTC) Quality of Life Questionnaire, Module 3 (QLQ-30) (cancer) and EORTC Breast Cancer, Module 23 (BR-23) surveys will also be provided to measure cancer-specific indices of quality of life. The FACIT Bone Pain (FACIT-BP) questionnaire will be used to examine bone pain, and the Brief Symptom Inventory (BSI-18) will be used to assess psychological distress for the anxiety, depression, somatisation and global distress severity domains. The Insomnia Severity Index (ISI) will be used to measure sleep quality disturbance, and the Godin Leisure-Time Exercise questionnaire will be applied to examine self-reported physical activity levels.

### Exercise programme

Participants assigned to the exercise arm will be required to participate in a modular, multi-modal exercise programme (M3EP), which includes spinal isometric training (SIT) for 12 weeks. The combined M3EP-SIT programme requires participants to attend three clinic-based exercise sessions each week spanning 60 min in duration (including warm up and cool down), supervised by an accredited exercise physiologist (AEP; Exercise and Sport Science Australia). Participants will also be asked to perform the SIT portion of the programme during two additional home-based exercise sessions each week spanning 15 min in duration. During combined M3EP-SIT sessions, spinal isometric training will be provided first, followed by the modular multi-modal exercise programme (Table 3).

**Table 3:** Weekly distribution of testing, M3EP and SIT exercise sessions across the exercise intervention

	Monday	Tuesday	Wednesday	Thursday	Friday	Saturday	Sunday
Week 0	Baseline testing						
Week 1 to Week 2	SIT	–	SIT	–	SIT	REST	REST
	M3EP		M3EP		M3EP		
Week 3 to Week 12	SIT	SIT	SIT	SIT	SIT	REST	REST
	M3EP		M3EP		M3EP		
Week 13	Post-Intervention Testing						

Clinic exercise sessions occur on Monday, Wednesday and Friday; home isometric exercise sessions occur on Tuesday and Thursday. Spinal isometric exercises are provided at the start of all clinic exercise sessions (following a general warm up). Home-based SIT starts from week 3 onwards to enable appropriate familiarisation and training during the first 2 weeks of the programme

*Abbreviations*: *SIT* spinal isometric training (15 min), *M3EP* modular multi-modal exercise programme (60 min)

The M3EP component of the programme will comprise resistance, aerobic and flexibility exercises in accordance with Galvão and colleagues [48, 51, 55–58]. This M3EP component is designed to minimise loads on affected skeletal sites throughout the body. Exercise prescriptions for all activities will be modified based on the location and extent of bone metastases (Table 4). Resistance exercise will be set using repetition maximums (RM). Participants will be asked to perform six different resistance exercises using major muscle groups, subject to the location and extent of bone metastases, at 10–12 RM for three sets per exercise to achieve moderate intensity and volume. Aerobic exercise will be set using age-predicted heart rate maximum (HRmax). Participants will engage in cardiovascular exercise using various modes including treadmill, cycling and rowing ergometers, performed at 60–85% HRmax for 20–30 min using heart rate monitors (Polar Electro Oy, Finland). Flexibility exercise will involve static stretching of muscles at all joints considered important for function, and for all muscles engaged during the session. All stretches will involve 2–4 sets per muscle group with a 30–60 s hold per set.

**Table 4:** Modular multi-modal exercise programme for patients with bone metastases [48, 51, 56, 57]

Metastasis site	Resistance			Aerobic		Flexibility
	Upper	Trunk	Lower	WB	NWB	Static
Pelvis	√	√	√**		√	√
Lumbar spine	√		√		√	√***
Thoracic spine/ribs	√*		√	√	√	√***
Proximal femur	√	√	√**		√	√
All regions	√*		√**		√	√***

*Abbreviations*: *WB* weight bearing (e.g. walking), *NWB* non-weight bearing (e.g. cycling)

√ represents target exercise region

*Exclusion of shoulder flexion/extension/abduction/adduction - inclusion of elbow flexion/extension

**Exclusion of hip extension/flexion - inclusion of knee extension/flexion

***Exclusion of spine/flexion/extension/rotation

The SIT component of the programme will comprise exercises that isometrically load deep spinal muscles as well as the larger superficial trunk musculature. These will be performed five times per week. Three sessions will be supervised by an AEP with the M3EP component at an exercise clinic, with an additional two sessions self-managed by the participant. This SIT component is designed to directly target and stimulate spinal lesion site(s) through muscular contraction, thus isometric exercises have been designed to activate the full spinal column due to the commonality of lesions in cervical, thoracic and lumbar regions, the feasibility of which has been demonstrated [59]. The SIT programme will require the participants to perform five exercises in whole and partial weight-supported prone and supine positions on the floor, whilst maintaining a neutral spine position (isometrically) during gentle and dynamic accessory movements. If floor exercises are contraindicated for the patient due to physical restrictions, alternate seated and standing isometric exercises will be provided. All patients will be initially provided with familiarisation of breathing technique, trunk stabilisation and hip control. Basic spinal isometric exercises will first be used to ensure safe and correct technique prior to progressing to intermediate or more challenging exercises, which include less stability or dynamic accessory movements [89]. Isometric progression of patients from beginner to advanced exercises will be individually determined on the basis of their physical capabilities and known contraindications. An assortment of spinal isometric exercises canvassing beginner to advanced and floor to standing are described in Additional files 1 and 2, respectively, with photographic demonstration of prone and supine exercises in Additional file 3.

### Statistical analysis

Data will be analysed using SPSS (IBM Corporation; Chicago, IL, USA). Normality of distribution of continuous variables will be determined by Shapiro-Wilk test and visual inspection of the data. Analyses will include standard descriptive characteristics, the *t* test and two-way (group × time) repeated measures analysis of variance (ANOVA) (or analysis of covariance as appropriate) to examine differences between groups over time. Any data that are not normally distributed will be log-transformed or non-parametric tests will be used. The Pearson chi-square test will be used to analyse categorical variables. An alpha level of *p* ≤ 0.05 will be applied to establish statistical significance. Effect sizes will also be calculated in accordance with Hopkins [90]: *d* ≥ 0.2 is small; *d* ≥ 0.6 is moderate; *d* ≥ 1.2 is large; *d* ≥ 2.0 is very large. Incomplete data and missing values will be primarily managed using an intention-to-treat approach [91] with multiple imputation, specifically using maximum likelihood imputation of missing values. To ensure the robustness of our findings, a secondary sensitivity analysis [92, 93] will be conducted using a complete-cases approach.

### Dissemination plan

Demonstrating the feasibility and safety of delivering targeted exercise (controlled mechanical loads) to skeletal sites with osteolytic lesions in patients with advanced cancer will lead to potential changes in clinical practice. Accordingly, if proven to be safe and feasible, the outcomes of this pilot study will form the basis of future phase II and III clinical trials to establish efficacy; will be published in high-impact peer-reviewed journals; will be presented at national and international conferences or research meetings and will be delivered to the community, consumer-led forums, local hospital departments and university seminars. Evidence from this pilot study may contribute to the renewal of current clinical exercise oncology guidelines for patients with cancer, specifically those in the advanced stages with a high disease burden, within national and international exercise and oncology associations.

Last, the National Breast Cancer Foundation is a charitable organisation dedicated to improving patient outcomes for women and men with breast cancer. As the funder of this pilot study, the National Breast Cancer Foundation will assist with disseminating study outcomes through their extensive clinical, academic and consumer networks nationally. Similarly, the Cancer Council of Western Australia is the premier cancer charity in Western Australia, with wide-reaching connections to clinicians, academics, cancer survivors and their families, and will also assist in disseminating study outcomes through their state-wide networks.

### Patient and public involvement

The Exercise Medicine Research Institute engages consumer representatives (patients with cancer and their families) throughout the conceptual design and development of its research programme to ensure all research questions directly address the needs of patients (in this case, patients with advanced breast cancer), including the engagement of prospective trial participants in a respectful, ethical and impactful way. During the development of this study protocol, authors NHH and RUN presented at national and state breast cancer meetings to patients and clinicians and sought feedback to confirm and optimise the study design. The National Breast Cancer Foundation (the funder of the study), and other cancer charities and associations (such as Breast Cancer Network Australia, Breast Cancer Care Western Australia and the Cancer Council of Western Australia) will assist in the dissemination of findings to their cancer support groups and the general public. Study participants will receive their individual results at the conclusion of their involvement and overall study results at the conclusion of the study.

The research team of this study protocol includes a surgical oncologist (CS), radiation oncologists (NAS, RC) and medical oncologists (KTF, DT, HM, TC, ADR) who work with the target population on a daily basis, from which patient priorities, experiences and preferences gleaned from this engagement helped inform the development of the research questions and outcome measures. Last, the broader research team (NHH, RUN, DAG, DRT, NAS) have conducted research studies in exercise oncology involving a large number of patients over the course of the past 15 years, where participants have provided feedback to investigators to help design feasible, safe and effective exercise oncology clinical trials. This sizeable interaction across the clinical and community landscape contributed substantially to the design of this project.

## Discussion

Complications arising from bone metastases present a major clinical issue for patients and clinicians alike [18], with bone metastases evident in over 80% of metastatic breast carcinoma (advanced breast cancer) cases. Currently, it is a treatable, yet incurable stage of disease, thus strategies that delay disease progression and extend survival without an adverse impact on quality of life or excessive clinical risk are highly sought after. Exercise medicine is an emerging field in oncology (i.e. exercise oncology), known for its neo-adjuvant and adjuvant role for symptom control, reduction of treatment toxicity and ability to improve the tolerance of and recovery from intensive cancer treatment regimens [48, 52, 65, 94–103]. Recent insights are beginning to illustrate the synergistic (assistive) and targeted (independent) role of exercise medicine in patients with cancer throughout the disease trajectory to assist with delaying disease progression and plausibly extending overall survival, largely through preclinical studies ([13, 14, 47–51, 70–74, 83, 104–118]) or epidemiological associations [119–134]. Most promisingly, exercise seems to be able to favourably modulate tumour biology towards improving cancer control, including skeletal orthotopic models, highlighting a key candidate intervention for human, patient-focused studies to target and pursue [14, 51, 135].

Exercise medicine may positively alter tumour biology through numerous mechanical and non-mechanical mechanisms targeting local, neighbouring and systemic pathways in response to various modes and dosages of activity [70–74, 105, 110, 112, 113, 136]. Specifically, exercise regulates endocrine-paracrine activity, systemic immune function (pro-inflammatory and anti-inflammatory activity), blood glucose and blood cholesterol levels, insulin response and body composition [97–101, 106, 116, 137, 138]. Exercise can also epigenetically modulate tumour vasculature (morphology and permeability), tumour cell proliferation (growth and distribution), telomeres (length and enzyme activity), platelet functions (cloaking and adhesion) and oxidative stress capacity [49, 50, 113, 139–144]. Whilst exercise concurrently acts across many of these regulatory and modulatory pathways, the targeted suppression of tumour formation, growth and invasion through mechanically driven epigenetic alterations, and muscle driven endocrine-paracrine activity is of particular interest. Indeed, biological alterations from biomechanical stimuli and biochemical responses (an emerging field known as “mechanomics”, applied to exercise oncology) [8, 12, 118, 145, 146] presents clinicians and allied health practitioners (such as clinical exercise physiologists) with a unique opportunity to suppress the growth and spread of metastatic breast carcinoma in bone through targeted exercise interventions.

Compelling new insights from metastatic orthotopic animal models demonstrate the ability of mechanical stimulation (i.e. repeated bouts of external compression) to interfere with tumour-driven remodelling in skeletal tissue containing human breast cancer cells [13, 41]. Exercise is a dose-dependent mechanical stimulant (with evidence of dose-response) that can be safely prescribed to patients with advanced prostate cancer and sclerotic metastases [14, 48, 51, 55, 57, 58, 106]. It is of equal interest to explore whether this can also be achieved in patients with advanced breast cancer with osteolytic metastases, who experience skeletal fragility at much faster rates than their counterparts with sclerotic metastases. Furthermore, it is of interest to explore whether skeletal integrity and tumoural suppression are prevalent in humans, as preclinical studies do not always translate to the human condition [61–63]. Indeed, it is not yet known whether disease-affected bone sites adapt to mechanical stimuli to the same order of magnitude or in the same morphological manner as unaffected healthy bone sites; nor is it clear how muscle surrounding lesions may adapt given the catabolic tumour microenvironment.

Accordingly, this study is our evaluation of the feasibility, safety and preliminary efficacy of a modular, multi-modal exercise programme, coupled with spinal isometric training, to provide a non-invasive, low-cost, innovative and scalable therapy in the management of advanced breast cancer. Examination of the modulatory potential of direct and targeted mechanical loading of osteolytic spinal bone metastases will also be conducted by quantifying tumour morphology and systemic activity of metastatic biomarkers (HIF-1α, local hypoxia; TGF-β, transformation growth-like factor), whilst also exploring whether targeted exercise can reduce bone pain, preserve neighbouring skeletal mass and structure (i.e. unaffected vertebrae above and below affected lesion sites) and reduce or avoid exacerbation of bone pain. Additionally, this study will also examine the multifaceted and broader effects of exercise participation by patients with advanced breast cancer on muscle and bone health (mass and strength), adiposity (subcutaneous and visceral), physical fitness and function and psychosocial health (focusing on quality of life).

Outcomes of this study will inform future research into sclerotic, osteolytic or mixed lesions across solid and haematological malignancies (such as multiple myeloma), particularly in patients with cancer and extensive or widespread bone metastases and high disease burdens who would otherwise be contraindicated for exercise. Optimistically, the generalisability of this exercise programme across models seems achievable, given it is supervised, individualised and tailored to each patient’s unique condition, and thus auto-regulated accordingly [48, 51]. The mechanistic insights of this study may also inform the development of effective pharmaceutical or medical treatments with avenues to target bone metastases. Given the preliminary efficacy (phase 1) nature of this pilot study, the results will be used to pursue larger phase II and III clinical trials to determine the efficacy of the programme in tumour suppression or regression in patients with osteolytic bone metastases secondary to breast cancer, and to develop an exercise program that can inevitably and immediately be delivered in clinical and community settings by accredited or certified clinical exercise physiologists.

## Additional files

Additional file 1: Floor-based, spinal isometric exercise library for patients with prostate cancer and spinal bone metastases, to cater for varying physical capabilities and training progression rates. (PDF 554 kb)

Additional file 2: Seated and standing, spinal isometric exercise library for patients with prostate cancer and spinal bone metastases, to cater for patients unable to perform floor-based exercises. (PDF 511 kb)

Additional file 3: Photographic examples of prone (left) and supine (right) floor-based spinal isometric exercises, illustrating the start position and final hold positions of each labelled exercise to assist exercise physiologists and patients with cancer. The patient shown has signed a media release consent form. (PDF 923 kb)

Additional file 4: SPIRIT 2013 Checklist: Recommended items to address in a clinical trial protocol and related documents. (DOCX 51 kb)

## Abbreviations

aBMC: Areal bone mineral content
aBMD: Areal bone mineral density
ACTRN: Australian Clinical Trials Registry Number
ADR: Andrew D Redfern
AEP: Accredited exercise physiologist
ALP: Alkaline phosphatase
ANOVA: Analysis of variance
ANZCTR: Australia and New Zealand Clinical Trials Registry
BA: Bone area
BMI: Body mass index
BP: Bone pain
BR-23: Breast Cancer, Module 23
BSI-18: Brief Symptom Inventory, Module 18
CRP: C-reactive protein
CS: Christobel Saunders
Ct.Th: Cortical thickness
Ct.vBMD: Cortical volumetric bone mineral density
CTCAE: Common terminology criteria for adverse events
DAG: Daniel A Galvão
DRT: Dennis R Taaffe
DT: Daphne Tsoi
DXA: Dual-energy x-ray absorptiometry
ECOG: European Cooperative Oncology Group
ECU: Edith Cowan University
Fa.Ar: Fat area
FACIT: Functional Assessment of Chronic Illness Therapy
FL.Ab: Fracture load, absolute
FL.Rel: Fracture Load, Relative
HIF-1α: Hypoxia-inducible factor, one alpha.
HM: Hilary Martin
HR+: Hormone receptor positive.
HR-: Hormone receptor negative
HREC: Human Research Ethics Committee
HRmax: Heart rate maximum
ICMJE: International Committee of Medical Journal Editors
ID: Identifier
IQOLA: International Quality of Life Assessment
ISI: Insomnia Severity Index
KTF: Kynan T Feeney
M3EP: Modular, multimodal exercise program
Ma.Ar: Marrow area
Ma.vBMD: Marrow volumetric bone mineral density
MRI: Magnetic resonance imaging
Mu.Ar: Muscle area
Mu.Den: Muscle density
NAS: Nigel A Spry
NHH: Nicolas H Hart
NTx: Amino-terminal collagen type-I telopeptide
P1NP: Amino-terminal propeptide of type 1 procollagen
pQCT: Peripheral quantitative computed tomography
QLQ-30: Quality of Life Questionnaire, Module 3
RC: Raphael Chee
RM: Repetition maximum
RUN: Robert U Newton
SCGOPHG: Sir Charles Gairdner Osborne Park Health Group
SF-36: Short Form, Module 36
SIT: Spinal isometric training
SJOG: St John of God Hospital
SKC: Suzanne K Chambers
SPSS: Statistical Package for the Social Sciences
SSIPOL: Stress-Strain Index Polar
Tb.Ar: Trabecular area
Tb.vBMD: Trabecular volumetric bone mineral density
TC: Tim Clay
TGF-β: Transformation growth-like factor, beta.
Tt.Ar: Total area
Tt.vBMD: Total volumetric bone mineral density
VAS: Visual analogue scale
VAT: Visceral adipose tissue
