# Supplementary material for: Mechanical suppression of osteolytic bone metastases in advanced breast cancer patients: a randomised controlled study protocol evaluating safety, feasibility and preliminary efficacy of exercise as a targeted medicine
Source: Trials. 2018 Dec 20;19:695. doi: 10.1186/s13063-018-3091-8 (PMC6302473; doi:10.1186/s13063-018-3091-8)
Supplement: Supplementary file 1 — Floor-based, spinal isometric exercise library for patients with prostate cancer and spinal bone metastases, to cater for varying physical capabilities and training progression rates. (PDF 554 kb) [file 13063_2018_3091_MOESM1_ESM.pdf]

Supplementary Table 1. Floor-based, spinal isometric exercise library for breast cancer patients with spinal bone metastases to cater for varying physical capabilities and training progression rates.

Exercise	Instruction
Abdominal Brace #1:<br>- Single Leg Lift	**Start position:** Supine on floor with arms by their side, knees bent and feet on the floor.<br>**Instruction:** Pre-activate and set abdominals to hold a neutral spine position (isometric). Slowly raise one foot off the floor until shank is parallel to the floor. Hold for 10 seconds. Lower foot to floor and repeat on the other limb.<br>**Volume:** Perform 2-4 sets of 8-12 repetitions.<br>**Rest:** 60 seconds between sets.
Abdominal Brace #2:<br>- Double Leg Lift	**Start position:** Supine on floor with arms by their side, knees bent and feet on the floor.<br>**Instruction:** Pre-activate and set abdominals to hold a neutral spine position (isometric). Slowly raise both feet off the floor until both shanks are parallel to the floor. Hold for 10 seconds. Lower both feet to floor.<br>**Volume:** Perform 2-4 sets of 8-12 repetitions.<br>**Rest:** 60 seconds between sets.
Abdominal Brace #3:<br>- Leg Fall Out	**Start position:** Supine on floor with hands resting on hips, knees bent and feet on the floor.<br>**Instruction:** Pre-activate and set abdominals to hold a neutral spine position (isometric). Slowly allow one leg to rotate outward towards the floor by relaxing the adductor muscles. Do not allow rotation at the spine or pelvis. Repeat on opposite leg.<br>**Volume:** 2-4 sets of 8-12 repetitions<br>**Rest:** 15 seconds between sets.
Abdominal Brace #4:<br>- Foot Slide	**Start position:** Supine on floor with hands resting on hips, knees bent and feet on the floor.<br>**Instruction:** Pre-activate and set abdominals to hold a neutral spine position (isometric). Slowly slide one leg out (extending at the hip and knee) and back along the floor. Repeat on opposite leg.<br>**Volume:** 2-4 sets of 8-12 repetitions<br>**Rest:** 15 seconds between sets.
'All Fours' Position #1:<br>- Hip Hinges	**Start position:** Prone, resting on knees, with outstretched hands beneath the shoulders.<br>**Instruction:** Pre-activate and set abdominals to hold a neutral spine position (isometric). Slowly flex at the hips and knees, moving backward. Patient should sit into this position so that their buttocks touches their heels while arms remain fixed. Return to start position.<br>**Volume:** 2-4 sets of 8-12 repetitions<br>**Rest:** 15 seconds between sets.
'All Fours' Position #2:<br>- Arm Extension	**Start position:** Prone, resting on knees, with outstretched hands beneath the shoulders.<br>**Instruction:** Pre-activate and set abdominals to hold a neutral spine position (isometric). Lift one arm off the ground. Maintain balance by holding a neutral and firm spine. Pause for 5 seconds before slowly lowering back to the floor. Repeat on alternate arm.<br>**Volume:** 2-4 sets of 8-12 repetitions<br>**Rest:** 30 seconds between sets.
'All Fours' Position #3:<br>- Leg Extension	**Start position:** Prone, resting on knees, with outstretched hands beneath the shoulders.<br>**Instruction:** Pre-activate and set abdominals to hold a neutral spine position (isometric). Lift one leg off the ground. Maintain balance by holding a neutral and firm spine. Pause for 5 seconds before slowly lowering back to the floor. Repeat on alternate leg.<br>**Volume:** 2-4 sets of 8-12 repetitions<br>**Rest:** 30 seconds between sets.
'All Fours' Position #4:<br>- Arm & Leg Extension	**Start position:** Prone, resting on knees, with outstretched hands beneath the shoulders.<br>**Instruction:** Pre-activate and set abdominals to hold a neutral spine position (isometric). Lift one arm and the opposite leg off the ground. Maintain balance by holding a neutral spine. Pause for 5 seconds before slowly lowering back to the floor. Repeat on the alternate arm/leg combination.<br>**Volume:** 2-4 sets of 8-12 repetitions.<br>**Rest:** 30 seconds between sets.
Bridge #1:<br>- Full Hip Extension	**Start position:** Supine on floor with hands by their side, knees bent and feet on the floor.<br>**Instructions:** Pre-activate and set abdominals to hold a neutral spine position (isometric). Lift hips to align with shoulders and knees. Pause for 5 seconds in this position prior to slowly lowering hips back to the floor.<br>**Volume:** 2-4 sets of 8-12 repetitions.<br>**Rest:** 60 seconds between sets.

Bridge #2:<br>- Single Hip Extension	**Start position:** Supine on floor with hands by their side, knees bent and feet on the floor.<br>**Instructions:** Pre-activate and set abdominals to hold a neutral spine position (isometric). Lift hips to align with shoulders and knees. Once in this position, slowly extend and straighten one leg. Slowly return extended leg back to the floor before also lowering the hips back to the floor. Alternate between legs.<br>**Volume:** 2-4 sets of 8-12 repetitions.<br>**Rest:** 60 seconds between sets.
Bridge #3:<br>- Side Bridges	**Start position:** Lying on their side, with arm abducted, resting on their forearm.<br>**Instructions:** Pre-activate and set abdominals to hold a neutral spine position (isometric). Lift hips to align with shoulders and knees. Maintain knee contact with the floor. Hold this position for up to 90 seconds (or as long as achievable without compromising technique). Alternate sides. This can be progressed by performing this exercise with legs fully extended (resting on forearm and feet).<br>**Volume:** Hold for 10- 90 seconds. Repeat 2-4 times.<br>**Rest:** 60 seconds between sets.
Bridge #4:<br>- Prone Planks	**Start position:** Prone, resting on knees, with outstretched hands beneath the shoulders.<br>**Instructions:** Pre-activate and set abdominals to hold a neutral spine position (isometric). Straighten legs while arms remain outstretched, ensuring abdominals remain braced. Hold this position for up to 90 seconds (or as long as achievable without compromising technique). This can be progressed by performing this exercise while resting on forearms.<br>**Volume:** Hold for 10-90 seconds. Repeat 2-4 times.<br>**Rest:** 60 seconds between sets.

Note: Always maintain neutral spine and control pelvic tilt. Spine must be supported through abdominal bracing to ensure safe delivery of these exercises [14,89]. Practitioners must always be cautious and observant to ensure correct technique and posture is upheld. Patients with cervical bone metastases are contraindicated for Bridge #1 or Bridge #2 and should not perform these two exercises.
