# Supplementary material for: Mechanical suppression of osteolytic bone metastases in advanced breast cancer patients: a randomised controlled study protocol evaluating safety, feasibility and preliminary efficacy of exercise as a targeted medicine
Source: Trials. 2018 Dec 20;19:695. doi: 10.1186/s13063-018-3091-8 (PMC6302473; doi:10.1186/s13063-018-3091-8)
Supplement: Supplementary file 2 — Seated and standing, spinal isometric exercise library for patients with prostate cancer and spinal bone metastases, to cater for patients unable to perform floor-based exercises. (PDF 511 kb) [file 13063_2018_3091_MOESM2_ESM.pdf]

Supplementary Table 2. Seated and standing, spinal isometric exercise library for breast cancer patients with spinal bone metastases to cater for patients unable to perform floor-based exercises.

Exercise	Instruction
Seated Exercise #1<br>- Marching	**Start position:** Seated on bench or fit-ball; back straight, knees bent and feet on floor.<br>**Instruction:** Pre-activate and set abdominals to hold a neutral spine position (isometric). Lift one knee up as high as flexibility allows (while maintaining upright posture). Pause at the top for 2 seconds and lower back down. Repeat with the opposite limb. Only one foot should be on the ground at a time to simulate slow paced marching. Focus on patient posture and trunk stability to ensure proper muscle activation. To increase difficulty,<br>**Volume:** Perform 2-4 sets of 8-12 repetitions (each leg).<br>**Rest:** 30 seconds between sets.
Seated Exercise #2<br>- Leg Extension	**Start position:** Seated on bench or fit-ball; back straight, knees bent and feet on floor.<br>**Instruction:** Pre-activate and set abdominals to hold a neutral spine position (isometric). Slowly raise one foot off the floor to straighten the leg (until the shank is parallel to the floor). Hold for 5 seconds. Lower the foot to the floor. Repeat on the other leg. To increase difficulty, if seated on a stable bench (or chair), patient can use both legs simultaneously. Alternatively, patients can perform single-leg extensions against theraband resistance or while seated on a fit-ball. Focus on patient posture and trunk stability to ensure proper muscle activation.<br>**Volume:** Perform 2-4 sets of 8-12 repetitions (each leg).<br>**Rest:** 30 seconds between sets.
Seated Exercise #3<br>- Theraband Row	**Start position:** Seated on bench or fit-ball; back straight, knees bent and feet on floor.<br>**Instruction:** Pre-activate and set abdominals to hold a neutral spine position (isometric). Wrap an elastic band around a fixed object at chest level in front of the patient. Use a band which provides a mild resistance. With arms fully extended, slowly pull the band towards the chest, keeping the elbows low and close to the body, until the elbows bend to approximately 90 degrees. Hold position for 5 seconds. Slowly return to the start position. Focus on patient posture and trunk stability to ensure proper muscle activation.<br>**Volume:** 2-4 sets of 8-12 repetitions<br>**Rest:** 30 seconds between sets.
Hybrid Exercise #1<br>- Sit-to-Stand	**Start position:** Seated on a bench or chair; back straight with arms crossed over.<br>**Instruction:** Pre-activate and set abdominals to hold a neutral spine position (isometric). Stand up from the seated position. Once fully upright, return slowly to a seated position. Aim is to perform this exercise without using arms for assistance (if possible). Avoid this exercise if patient has knee pathology. Focus on patient posture and **movement quality** to ensure proper muscle activation and safe performance of this exercise.<br>**Volume:** 2-4 sets of 8 repetitions<br>**Rest:** 60 seconds between sets.
Standing Exercise #1<br>- Wall Push-Up	**Start position:** Standing. Place hands on wall at shoulder height, with arms extended.<br>**Instruction:** Pre-activate and set abdominals to hold a neutral spine position (isometric). Slowly lower their body and hips towards the wall by bending at the elbows in a controlled manner. Once their face is near the wall, hold for 5 seconds and then slowly push against the wall to extend the arms and return back to starting position. Focus on patient posture and trunk stability to ensure proper muscle activation. To increase difficulty, patient can increase angle against the wall.<br>**Volume:** 2-4 sets of 8-12 repetitions<br>**Rest:** 30 seconds between sets.
Standing Exercise #2<br>- Wall Plank	**Start position:** Standing. Leaning forward on forearms against a wall.<br>**Instruction:** Pre-activate and set abdominals to hold a neutral spine position (isometric). While leaning on forearms against the wall, slowly move feet backward to desired angle between the body and wall. Focus on placing body weight onto forearms while keeping trunk muscle engaged, torso straight with neutral spine. Focus on patient posture and trunk stability to ensure proper muscle activation.<br>**Volume:** 2-4 sets of 8-12 repetitions<br>**Rest:** 30 seconds between sets.

Standing Exercise #3<br>- Theraband Row	**Start position:** Standing; back straight, split-stance (one foot forward, one foot back).<br>**Instruction:** Pre-activate and set abdominals to hold a neutral spine position (isometric). Wrap an elastic band around a fixed object at chest level in front of the patient. Use a band which provides a mild resistance. With arms fully extended, slowly pull the band towards the chest, keeping the elbows low and close to the body, until the elbows bend to approximately 90 degrees. Hold position for 5 seconds. Slowly return to the start position. Focus on patient posture and trunk stability to ensure proper muscle activation.<br>**Volume:** 2-4 sets of 8-12 repetitions<br>**Rest:** 30 seconds between sets.

Note: Always maintain an upright trunk (straight back) with neutral spine and control pelvic position during all exercises. Spine must be supported through abdominal bracing to ensure safe delivery of these exercises [14,89]. Practitioners must always be cautious and observant to ensure correct technique and posture is upheld.
