# Supplementary material for: Mechanical suppression of osteolytic bone metastases in advanced breast cancer patients: a randomised controlled study protocol evaluating safety, feasibility and preliminary efficacy of exercise as a targeted medicine
Source: Trials. 2018 Dec 20;19:695. doi: 10.1186/s13063-018-3091-8 (PMC6302473; doi:10.1186/s13063-018-3091-8)
Supplement: Supplementary file 3 — Photographic examples of prone (left) and supine (right) floor-based spinal isometric exercises, illustrating the start position and final hold positions of each labelled exercise to assist exercise physiologists and patients with cancer. The patient shown has signed a media release consent form. (PDF 923 kb) [file 13063_2018_3091_MOESM3_ESM.pdf]

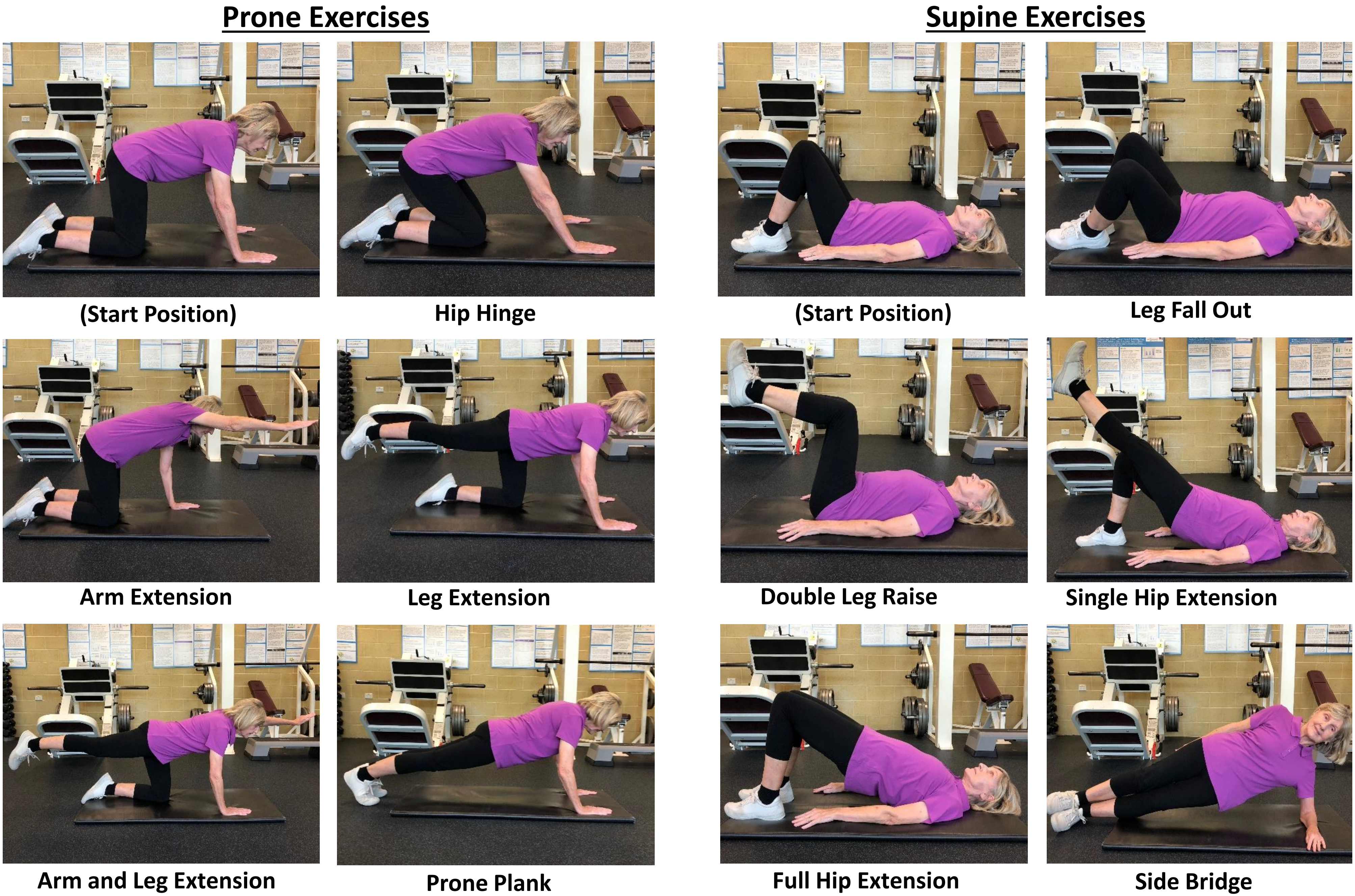

Supplementary Figure 3. Photographic examples of prone (left) and supine (right) floor-based spinal isometric exercises, illustrating the start position and final hold positions of each labelled exercise to assist exercise physiologists and cancer patients. The patient in-set was approached in order to obtain these photos explicitly for this manuscript, and has signed a media release consent form.
